# Supplementary material for: HIV-1 Drug Resistance Detected by Next-Generation Sequencing among ART-Naïve Individuals: A Systematic Review and Meta-Analysis
Source: Viruses. 2024 Feb 2;16(2):239. doi: 10.3390/v16020239 (PMC10893194; doi:10.3390/v16020239)
Supplement: Supplementary file 1 [file viruses-16-00239-s001.zip › Supplementary Figure S1-S6.pdf]

viruses

Supplementary Material

HIV-1 Drug Resistance Detected by Next-Generation Sequencing among ART-Naïve Individuals: A Systematic Review and Meta-Analysis

Fei Ouyang [1], Defu Yuan [1], Wenjing Zhai [1], Shanshan Liu [1], Ying Zhou [2,*] and Haitao Yang [1,3,*]

[1] Key Laboratory of Environmental Medicine Engineering of Ministry of Education, Department of Epidemiology and Health Statistics, School of Public Health, Southeast University, Nanjing 210009, China; 220213961@seu.edu.cn (F.O.); 230239083@seu.edu.cn (D.Y.); 220223638@seu.edu.cn (W.Z.); 220223703@seu.edu.cn (S.L.)
[2] Department of HIV/STD Control and Prevention, Jiangsu Provincial Center for Disease Control and Prevention, Nanjing 210009, China
[3] Jiangsu Health Development Research Center, Nanjing 210029, China
* Correspondence: yht@jscdc.cn (H.Y.); zhouy@jscdc.cn (Y.Z.)

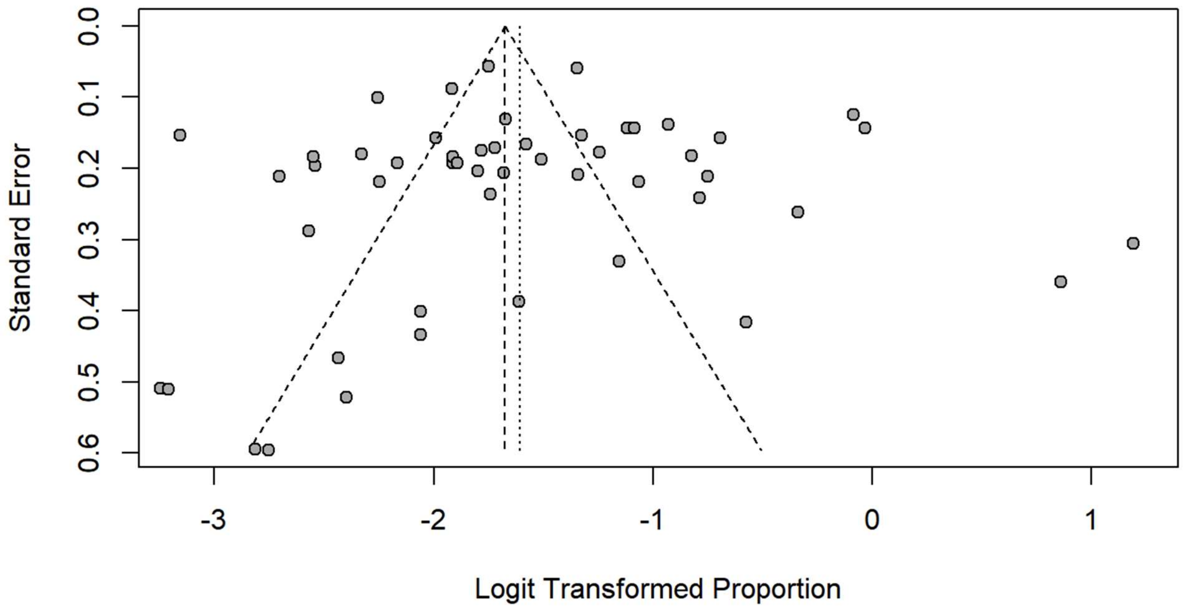

Figure S1**.** Funnel plot with pseudo 95% CI in this analysis.

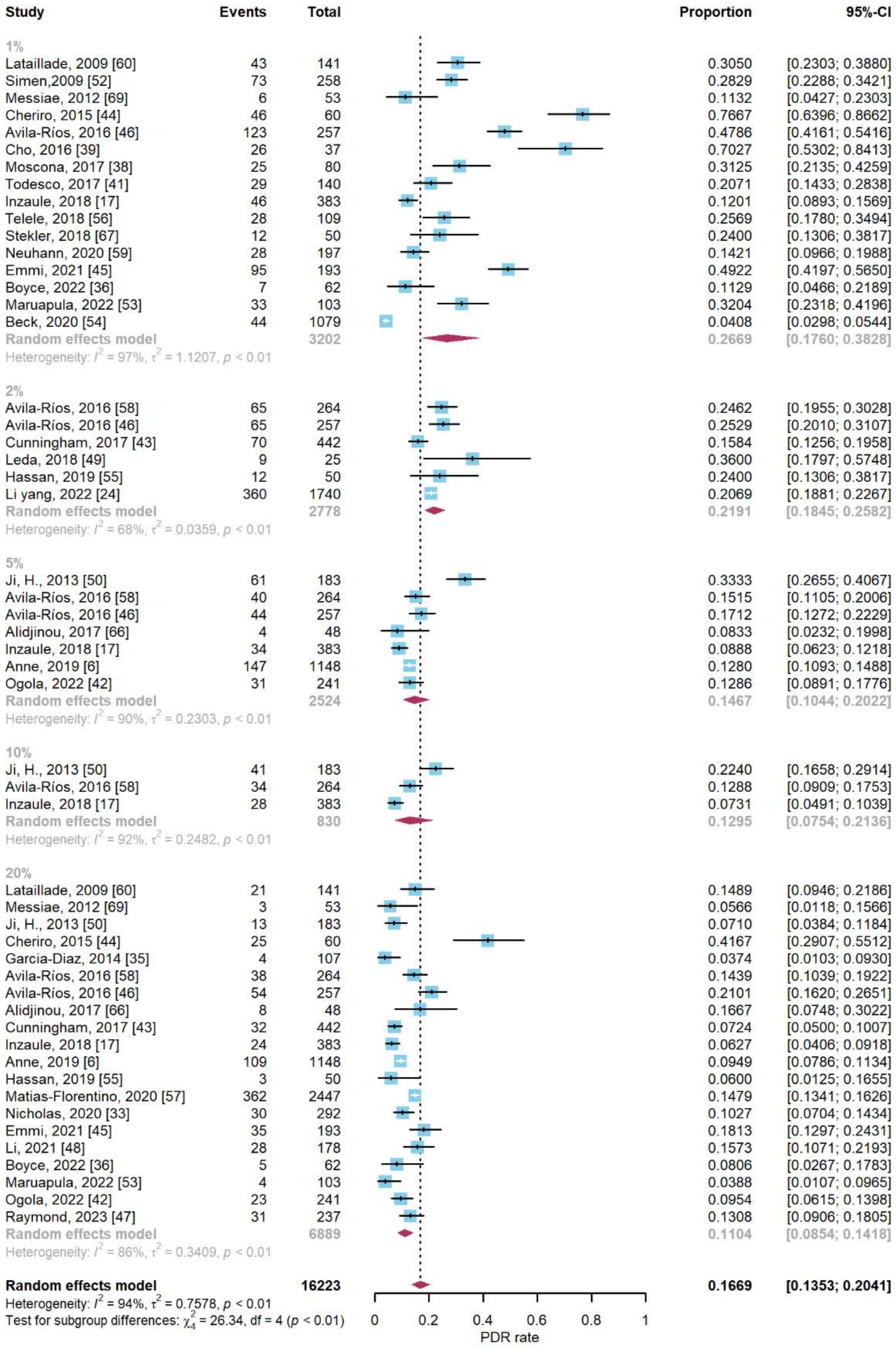

Figure S2. The overall prevalence of PDR at different threshold.

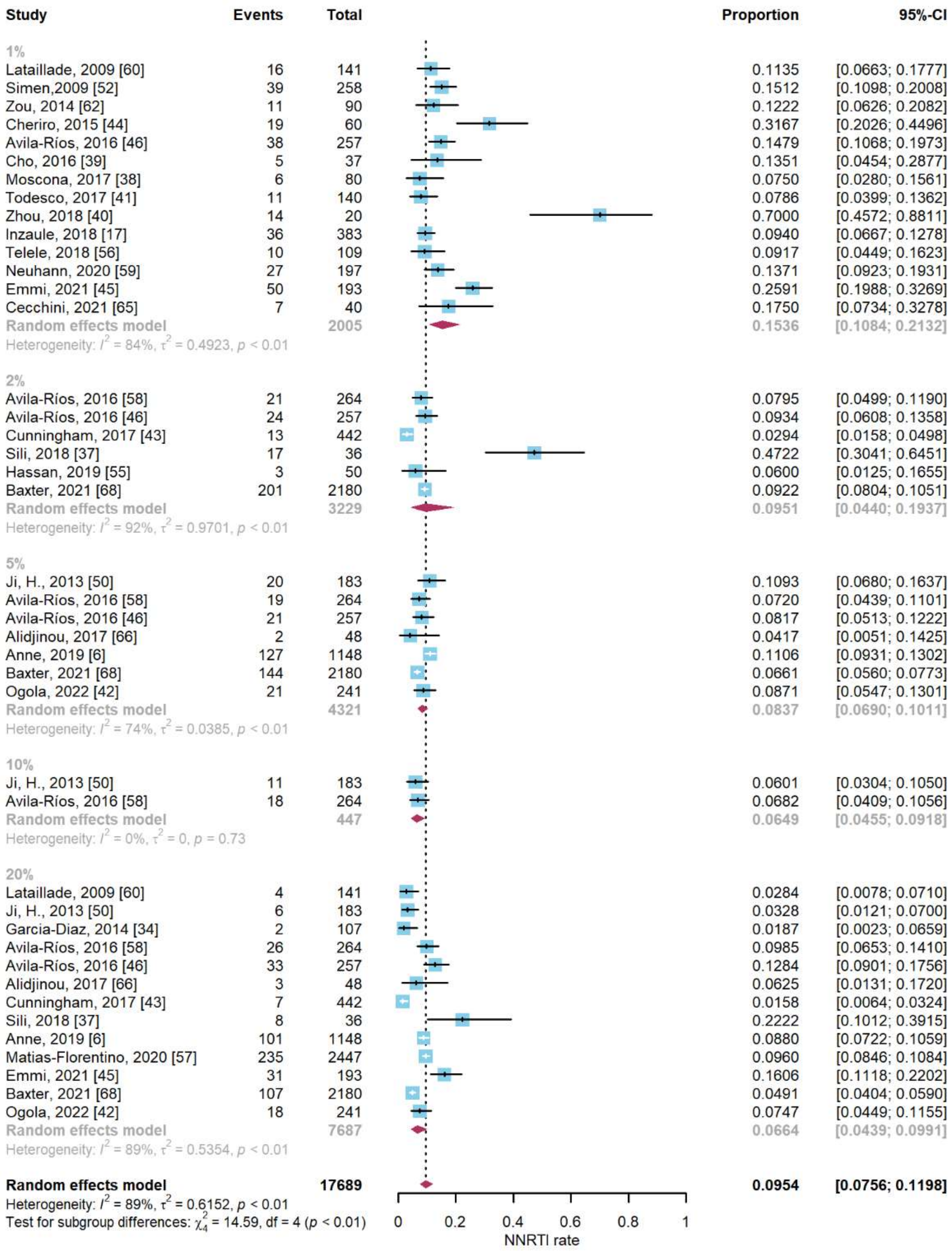

Figure S3. The overall prevalence of NNRTI at different threshold.

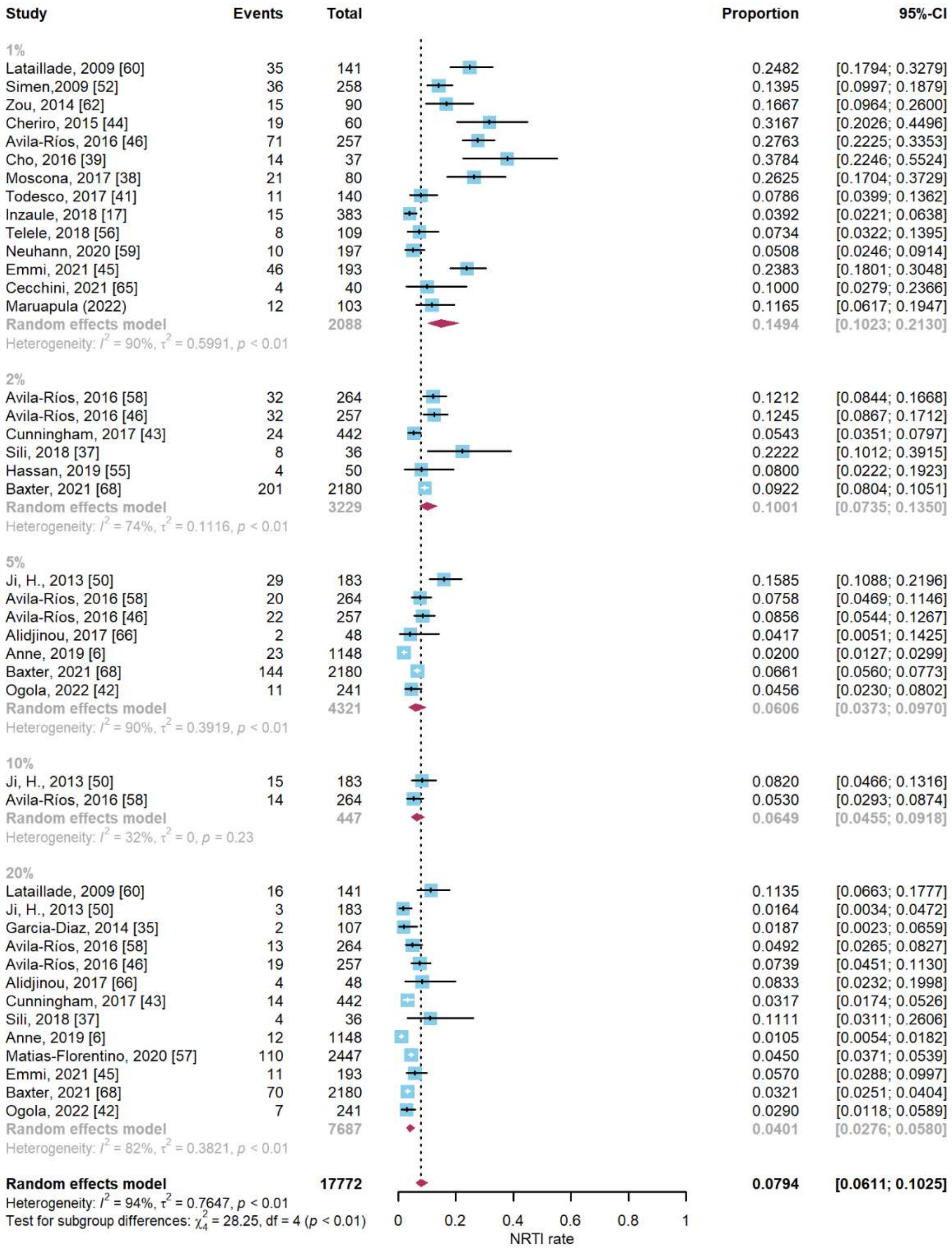

Figure S4. The overall prevalence of NRTI at different threshold.

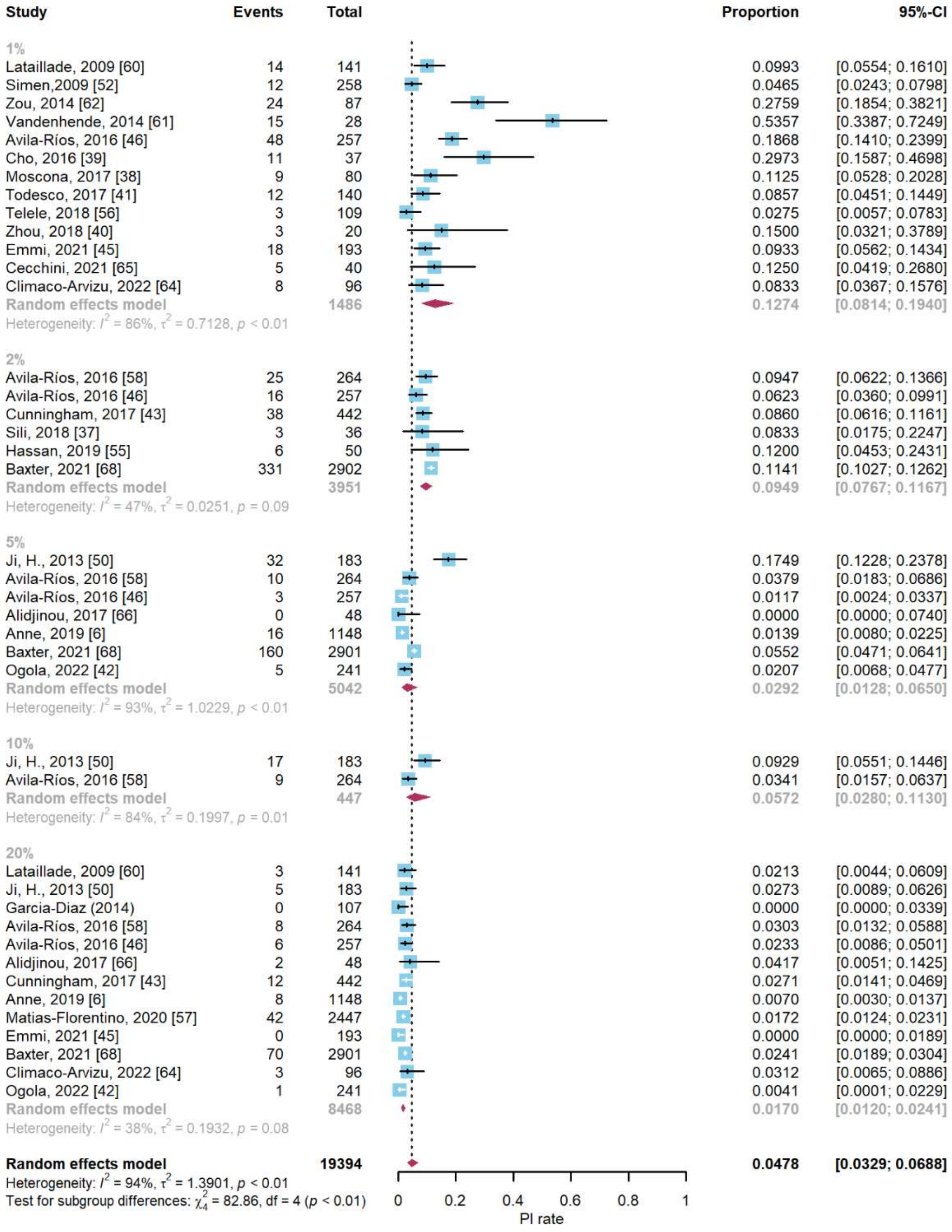

Figure S5. The overall prevalence of PI at different threshold.

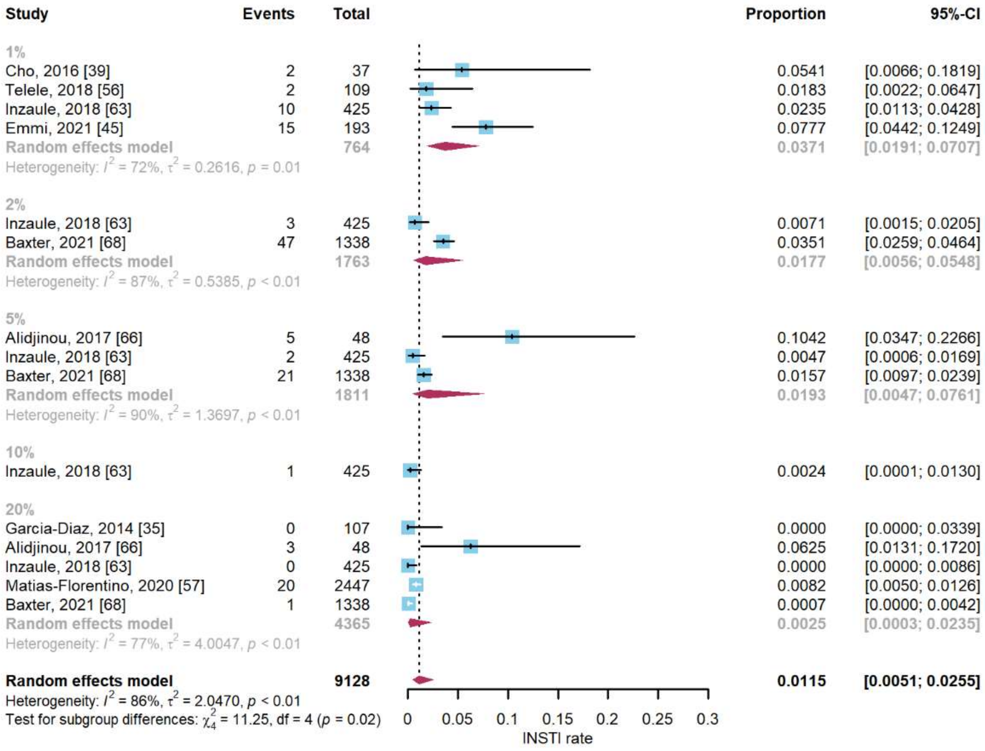

Figure S6. The overall prevalence of INSTI at different threshold.
