# Supplementary material for: HIV-1 Drug Resistance Detected by Next-Generation Sequencing among ART-Naïve Individuals: A Systematic Review and Meta-Analysis
Source: Viruses. 2024 Feb 2;16(2):239. doi: 10.3390/v16020239 (PMC10893194; doi:10.3390/v16020239)
Supplement: Supplementary file 1 [file viruses-16-00239-s001.zip › Supplementary Table S1.pdf]

Supplementary Material

HIV-1 Drug Resistance Detected by Next-Generation Sequencing among ART-Naïve Individuals: A Systematic Review and Meta-Analysis

Fei Ouyang [1], Defu Yuan [1], Wenjing Zhai [1], Shanshan Liu [1], Ying Zhou [2,*] and Haitao Yang [1,3,*]

[1] Key Laboratory of Environmental Medicine Engineering of Ministry of Education, Department of Epidemiology and Health Statistics, School of Public Health, Southeast University, Nanjing 210009, China; 220213961@seu.edu.cn (F.O.); 230239083@seu.edu.cn (D.Y.); 220223638@seu.edu.cn (W.Z.); 220223703@seu.edu.cn (S.L.)
[2] Department of HIV/STD Control and Prevention, Jiangsu Provincial Center for Disease Control and Prevention, Nanjing 210009, China
[3] Jiangsu Health Development Research Center, Nanjing 210029, China
* Correspondence: yht@jscdc.cn (H.Y.); zhouy@jscdc.cn (Y.Z.)

1 Supplementary Table

Table S1: The characteristics and literature quality scores of studies included in the meta-analysis.

Study	Geographic area	Year of sample collection	Sample size	Amplification success	Female（%）	Design	Age	Data report	NGS technology	Stage of HIV-1 infection at time of inclusion	Type of specimen	NGS thresholds	Study purpose	CD4 (cells/uL)	VL (log10 copies/mL)	The score of quality
Lataillade (2009)	Worldwide	——	148	141	34	case-control	——	SGS and NGS	454 pyrosequencing	Not specified	Plasma	<20%, 20%	TDR	Median:191	5.12 (3.48-5.86)	8
Simen (2009)	Not specified	——	264	258	15.9	cross-sectional	Mean: 38.5	SGS and NGS	454 pyrosequencing	(Mainly) chronic	Plasma	1%	LA-DRVs	Median(IQR)：206 (32-336)	Median(IQR): 5.35 (4.91-5.76)	6
Messiae (2012)	Europe	1999-2007	70	70	——	cohort	Median(IQR): 35 (29-41)	NGS	454 pyrosequencing	Not specified	Plasma	<20%, 20%	TDR	Median(IQR): 237(188-316)	Median(IQR): 2.3-5.0	5
Ji, H. (2013)	North America	——	183	183	——	cross-sectional	——	SGS and NGS	Pyrosequencing (Pyro-Mark)	Not specified	Serum	5%, 10%, 15%, 20%	LA-DRVs	——	——	5
Garcia-Diaz (2014)	Not specified	——	107	107	——	cross-sectional	——	SGS and NGS	454 pyrosequencing	Acute/recnet	Plasma	1%, 20%	TDR	——	Median(range):4.95 (3.14-6.33)	5
Vandenhende (2014)	Europe	2000.01-2009.06	29	28	20.7	cross-sectional	Median(IQR): 36.8 (33.0-39.4)	SGS and NGS	454 pyrosequencing	Not specified	Plasma	<20%, 20%	LA-DRVs	Median(IQR): 248(177-314)	Median(IQR): 5.0(4.6-5.2)	5
Zou (2014)	Asia	2010.07-2011.08	90	90	——	cross-sectional	Median(range): 37 (20-70)	NGS	454 pyrosequencing	Not specified	Plasma	1%	TDR	Median(range): 101.5 (8-332)	Median(range): 4.87 (3.60-6.19)	7
Cheriro (2015)	Africa	2009.09-2011.10	60	60	——	cross-sectional	—	NGS	454 pyrosequencing	Not specified	Plasma	1%, 20%	LA-DRVs	——	——	5
Avila-Ríos (2016)	North America	2015.02-2015.07	288	264	15	cross-sectional	Median(IQR):31 (24-39)	SGS and NGS	Illumina NGS	(Mainly) chronic	Plasma	2%, 5%, 10%, 20%	PDR	Median(IQR): 258 (86-428)	Median(IQR): 4.8 (4.2-5.3)	8
Avila-Ríos (2016)	Latin America	2011.08-2015.10	283	257	20.1	cohort	Median(IQR):31 (25-40)	SGS and NGS	Illumina NGS	(Mainly) chronic	Plasma	1%, 2%, 5%, 10%, 20%	PDR	Median(IQR):284 (92-459)	Median(IQR): 4.9 (4.2-5.5)	8
Cho (2016)	Asia	2011.01-2011.04	35	35	——	cross-sectional	——	NGS	454 pyrosequencing	(Mainly) chronic	Plasma	1%, 10%, 20%	PDR	——	Median(IQR): Donors: 4.64 (4.03-5.20), Chronic patients: 5.53 (5.00-5.35)	7
Moscona (2017)	Asia	2000-2014	80	80	20	cohort	Median(IQR):30.5 (18–45)	SGS and NGS	Illumina NGS	Acute/recnet	Plasma	1%, 5%	TDR	Median(IQR): 405 (310-579)	Median(IQR): 5.5 (4.6-6.2)	6
Todesco (2017)	Europe	——	140	140	0	cohort	——	SGS and NGS	454 pyrosequencing	Not specified	Plasma	<20%, 20%	TDR	Median(IQR):MSM: 498 (347-585); HSL: 348 (208-497)	Median(IQR):MSM: 4.9 (4.4-5.4); HSL: 4.9 (4.3-5.3)	7

Alidjinou (2017)	Europe	2013.10-2015.06	48	48	12.5	cross-sectional	Median(IQR): 36.5 (26.5–49.3)	SGS and NGS	454 pyrosequencing	Not specified	Plasma	5%. 20%	PDR	Median(IQR): 351.5 (172.5-493.8)	Median(IQR): 3.6 (3.2-4.0)	5
Cunningham (2017)	Europe	2011.07-2013.12	442	442	0	cross-sectional	Median(IQR): 32 (26-40)	NGS	Illumina NGS	Not specified	Plasma	2%, 2-20%, 20%	TDR	— —	— —	8
Inzaule (2018)	Africa	2007.03-2014.11	399	383	60.0	cohort	Median(IQR): 36.9 (31.0-42.8)	NGS	Illumina NGS	Not specified	Plasma	1%, 5%, 10%,20%	PDR	Median(IQR): 120 (53-192)	Median(IQR): 5.2 (4.5-5.7)	8
Sili (2018)	Asia	2013.01-2013.12	50	43	13.9	cross-sectional	Median(IQR): 32.5 (27-41)	NGS	454 pyrosequencing	Not specified	Plasma	2%-20%, 20%	TDR	— —	— —	6
Telele (2018)	Africa	2009.10-2011.12	109	109	55	cohort	Median: 30	SGS and NGS	Illumina NGS	Not specified	Plasma	1%	PDR	Mean±SD: 125±81	Mean±SD: 5.44±0.6	8
Zhou (2018)	Africa	2008.2-2008.6	20	20	— —	cross-sectional	— —	NGS	454 pyrosequencing	Not specified	Plasma	1-20%, 20%	LA-DRVs	— —	Range: 4.02-6.31	5
Inzaule (2018)	Africa	2007-2009	489	425	59.5	cohort	Median(IQR): 36.0 (30.0–42.0)	NGS	Illumina NGS	Not specified	Plasma	1%, 2%, 5%, 10%	PDR	Median(IQR): 119.0 (53.0-192.5)	— —	7
Mbunkah (2018)	Africa	2015-2016	360	298	63	cross-sectional	Median(range): 36 (18-68)	NGS	Illumina NGS	Not specified	Dried blood spot	15%, 3%-15%	TDR	Median(range): 303 (1-1112)	Median(range): 4.4 (3.7-5.9)	8
Leda (2018)	Latin America	2002-2009	25	25	— —	cohort	— —	NGS	454 pyrosequencing	Acute/recnet	Plasma	2%	LA-DRVs	— —	— —	5
Stekler (2018)	North America	1992-2010	50	50	50	cohort	Median(IQR): 29(24-33)	NGS	454 pyrosequencing	Acute/recnet	PBMCs	1%	TDR	— —	— —	5
Anne (2019)	Africa	2012.03-2016.06	1328	1148	70.3	cohort	Median(IQR): 32.9 (25.6-45.2)	NGS	Illumina NGS	Not specified	Plasma	5%, 20%	PDR	Median(IQR): 404 (262,559)	Median(IQR): 4.5 (3.9-5.2)	8
Hassan (2019)	Africa	2008-2013	50	50	72	cohort	Median(IQR): 34.7(28.9-42.0)	NGS	Illumina NGS	Not specified	Plasma	2%, <20%	PDR	Median(IQR): 348 (16-1076)	— —	5
Beck (2020)	Africa	2006, 2010, 2013.04	1231	1231	70.7	cohort	— —	NGS	Illumina NGS	Not specified	Plasma	2%	PDR	Median(IQR): 186 (102-278)	Median(IQR): 4.95 (4.23- 5.55)	6
Matias-Florentino（2020）	North America	2016.04-2018.06	2496	2447	4.3	cross-sectional	— —	NGS	Illumina NGS	Not specified	Plasma	20%	TDR	— —	— —	5
Nicholas (2020)	Asia	2012-2017	318	292	— —	cross-sectional	— —	NGS	Illumina NGS	Not specified	Plasma	<20%, 20%	LA-DRVs	— —	— —	5
Neuhann (2020)	Africa	2014-2015	200	197	61.9	cross-sectional	— —	NGS	Illumina NGS	Not specified	Serum	1%	PDR	Median(IQR):247.5(89-420)	Median(IQR):5.05(4.33-5.66)	8
Emmi (2021)	Europe	2017.01-2019.03	220	193	75.6	cross-sectional	— —	SGS and NGS	Illumina NGS	Not specified	Plasma	<5%, 5%-19%, 20%	PDR	330 (0-1200)	5.0 (1.7->7)	7
Baxter (2021)	Worldwide	2009.04-2013.12	2902	2901	27	cross-sectional	Median: 36	NGS	Illumina NGS	Not specified	Plasma	2%, 5%, 20%	TDR	Median: 643	Median: 4.3	7
Cecchini (2021)	Latin America	2008-2014	40	40	100	cohort	— —	NGS	Illumina NGS	Not specified	Plasma	1%	TDR	— —	— —	5
Li (2021)	Africa	2014.08-2018.07	178	178	64	case-cohort	Median(IQR): 32(27-42)	NGS	Illumina NGS	Not specified	Plasma	<20%, 20%	PDR	— —	— —	7
Boyce (2022)	North America	— —	69	66	100	case-cohort	Median(range): 25(14-44)	NGS	Illumina NGS	Not specified	Plasma	<20%, >20%	PDR	Median(range): 337 (1-917)	Median(range): 4.34 (2.73-5.92)	5

Li yang (2022）	Asia	2014-2015	1755	1740	14	cross-sectional	Median: 37	NGS	Illumina NGS	Not specified	Plasma	2%	TDR	– –	– –	7
Maruapula (2022)	Africa	2012.04-2015.04	108	103	99.1	cohort	Median (IQR): 27(24-31)	NGS	Illumina NGS	Not specified	Plasma	<20%, >20%	LA-DRVs	Median(IQR):365(225-497)	Median(IQR): 4.1(3.5-4.6)	7
Climaco-Arvizu (2022)	North America	2016-2019	100	96	1	cross-sectional	Median(range): 28(18-66)	NGS	Illumina NGS	Not specified	Plasma	1%, 20%	PDR	Median(IQR): 150 (68.0-355.78)	Median(IQR): 4.9(4.39-5.40)	5
Ogola (2022)	Africa	2016.01-2017.12	241	241	68.5	cross-sectional	Median(range): 37(16-61)	NGS	Illumina NGS	Not specified	Plasma	5%, 20%	PDR	Median(range): 245 (5–1,100)	Median(range): 28 (1.4–7.0)	6
Raymond（2023）	Europe	2018.01-2019.10	237	237	23	cohort	Mean ± SD: 41±11	NGS	Sentosa NGS system	Not specified	Plasma	3%, 20%	PDR	Mean±SD: 345±254	Mean±SD: 5.04±0.98	7

MSM, men who sex with men; HTS, Heterosexual; IDU, injective drug users; OGE, originating in countries with generalized epidemics; IQR, interquartile range; SD, standard deviation.
