# Supplementary material for: HIV-1 Drug Resistance Detected by Next-Generation Sequencing among ART-Naïve Individuals: A Systematic Review and Meta-Analysis
Source: Viruses. 2024 Feb 2;16(2):239. doi: 10.3390/v16020239 (PMC10893194; doi:10.3390/v16020239)
Supplement: Supplementary file 1 [file viruses-16-00239-s001.zip › Supplementary Table S2-S4.pdf]

Table S2. Compare the pretreatment drug resistance between 5% and 10% threshold.

Variables	Subgroup	Study	Event	Total	χ^2	P value
**PDR**						
	5%	7	361	2524	1.89	0.171
	10%	3	103	830		
**NNRTI**						
	5%	7	354	4321	1.59	0.207
	10%	2	29	447		
**NRTI**						
	5%	7	251	4321	0.34	0.561
	10%	2	29	447		
**PI**						
	5%	7	226	5042	1.67	0.196
	10%	2	26	447		
**INSTI**						
	5%	3	28	1811	4.62	0.032
	10%	1	1	425		

Table S3. Compare the pretreatment drug resistance between 2% and 5% threshold.

Variables	Subgroup	Study	Event	Total	χ^2	P value
**PDR**						
	2%	6	581	2778	39.57	<0.01
	5%	7	361	2163		
**NNRTI**						
	2%	6	279	3229	0.483	0.487
	5%	7	354	3967		
**NRTI**						
	2%	6	301	3229	33.65	<0.01
	5%	7	251	4070		
**PI**						
	2%	6	419	3951	124.72	<0.01
	5%	7	226	4816		
**INSTI**						
	2%	2	50	1713	6.96	0.008
	5%	3	28	1783		

Table S4: Compare the pretreatment drug resistance between 2% and 10% threshold.

Variables	Subgroup	Study	Event	Total	χ^2	P value
**PDR**						
	2%	6	581	2197	39.57	<0.01
	10%	3	103	727		
**NNRTI**						
	2%	6	279	2950	0.483	0.487
	10%	2	29	418		
**NRTI**						
	2%	6	301	2928	33.65	<0.01
	10%	2	29	447		
**PI**						
	2%	6	419	3532	124.72	<0.01
	10%	2	26	447		
**INSTI**						
	2%	2	50	1713	6.96	0.008
	10%	1	1	425		
