# Supplementary material for: HIV-1 Drug Resistance Detected by Next-Generation Sequencing among ART-Naïve Individuals: A Systematic Review and Meta-Analysis
Source: Viruses. 2024 Feb 2;16(2):239. doi: 10.3390/v16020239 (PMC10893194; doi:10.3390/v16020239)
Supplement: Supplementary file 1 [file viruses-16-00239-s001.zip › Supplementary Table S5-S8.pdf]

viruses

Supplementary Material

HIV-1 Drug Resistance Detected by Next-Generation Sequencing among ART-Naïve Individuals: A Systematic Review and Meta-Analysis

Fei Ouyang [1], Defu Yuan [1], Wenjing Zhai [1], Shanshan Liu [1], Ying Zhou [2,*] and Haitao Yang [1,3,*]

[1] Key Laboratory of Environmental Medicine Engineering of Ministry of Education, Department of Epidemiology and Health Statistics, School of Public Health, Southeast University, Nanjing 210009, China; 220213961@seu.edu.cn (F.O.); 230239083@seu.edu.cn (D.Y.); 220223638@seu.edu.cn (W.Z.); 220223703@seu.edu.cn (S.L.)

[2] Department of HIV/STD Control and Prevention, Jiangsu Provincial Center for Disease Control and Prevention, Nanjing 210009, China

[3] Jiangsu Health Development Research Center, Nanjing 210029, China

* Correspondence: yht@jscdc.cn (H.Y.); zhouy@jscdc.cn (Y.Z.)

Table S5. Frequency of drug resistance mutations in protease at different sensitivity thresholds.

PR	Classification	Surveillance	1-5%		5-10%		10-20%		>20%	
			n	%	n	%	n	%	n	%
L10F	Accessory	N	3	0.04	1	0.01	1	0.01	2	0.03
L10I	Other	N	53	0.70	14	0.18	18	0.24	318	4.18
L10R	Minor	N	10	0.13	1	0.01	0	0.00	0	0.00
L10V	Other	N	6	0.08	4	0.05	5	0.07	90	1.18
V11I	Other	N	14	0.18	3	0.04	5	0.07	12	0.16
K20I	Other	N	1	0.01	0	0.00	0	0.00	10	0.13
K20M	Other	N	1	0.01	1	0.01	0	0.00	16	0.21
K20R	Other	N	34	0.45	14	0.18	10	0.13	113	1.48
K20T	Minor	N	0	0.00	2	0.03	0	0.00	0	0.00
L23I	Minor	Y	27	0.35	0	0.00	1	0.01	0	0.00
L24I	Accessory	Y	4	0.05	0	0.00	0	0.00	1	0.01
L24M	Accessory	N	1	0.01	0	0.00	0	0.00	0	0.00
D30N	Major	Y	41	0.54	3	0.04	2	0.03	6	0.08
V32I	Major	Y	10	0.13	1	0.01	1	0.01	1	0.01
L33F	Minor	N	0	0.00	0	0.00	1	0.01	13	0.17
L33I	Minor	N	2	0.03	0	0.00	0	0.00	2	0.03
L33V	Other	N	3	0.04	2	0.03	1	0.01	11	0.14
E35G	Minor	N	1	0.01	0	0.00	0	0.00	1	0.01
M36I	Other	N	2	0.03	0	0.00	0	0.00	4	0.05
K43T	Accessory	N	2	0.03	2	0.03	1	0.01	8	0.11
M46I	Major	Y	57	0.75	13	0.17	10	0.13	31	0.41
M46L	Major	Y	13	0.17	3	0.04	3	0.04	17	0.22
M46V	Accessory	N	1	0.04	1	0.04	0	0.00	3	0.12
M46I/L	Major	Y	91	1.20	0	0.00	0	0.00	33	0.43
I47V	Major	Y	19	0.25	3	0.04	1	0.01	1	0.01
G48V	Major	N	2	0.03	0	0.00	0	0.00	0	0.00
I50L	Major	N	4	0.05	0	0.00	0	0.00	0	0.00
I50V	Major	Y	8	0.11	0	0.00	1	0.01	1	0.01
F53L	Accessory	Y	13	0.17	2	0.03	4	0.05	1	0.01
F53Y	Accessory	N	1	0.01	0	0.00	0	0.00	0	0.00
I54L	Major	Y	1	0.01	2	0.03	0	0.00	1	0.01
I54M	Major	Y	0	0.00	0	0.00	0	0.00	1	0.01
I54S	Major	N	1	0.01	0	0.00	0	0.00	0	0.00
I54T	Major	Y	5	0.07	1	0.01	2	0.03	0	0.00
I54V	Major	Y	2	0.03	0	0.00	1	0.01	9	0.12
Q58E	Accessory	N	1	0.01	0	0.00	0	0.00	52	0.68
D60E	Major	N	1	0.01	0	0.00	0	0.00	0	0.00
L63P	Other	N	3	0.04	0	0.00	0	0.00	6	0.08
L67V	Other	N	0	0.00	0	0.00	0	0.00	2	0.03
L68V	Accessory	N	4	0.05	2	0.03	3	0.04	1	0.01
A71I	Other	N	5	0.07	0	0.00	0	0.00	7	0.09
A71T	Other	N	32	0.42	8	0.11	11	0.14	301	3.95
A71V	Other	N	17	0.22	14	0.18	9	0.12	416	5.46
G73C	Accessory	Y	1	0.01	0	0.00	0	0.00	0	0.00
G73S	Accessory	Y	1	0.01	1	0.01	1	0.01	0	0.00
L74I	Other	N	2	0.03	0	0.00	2	0.03	78	1.02
L74M	Other	N	23	0.30	2	0.03	2	0.03	9	0.12
T74S	Other	N	11	0.14	0	0.00	0	0.00	7	0.09
L76V	Major	Y	2	0.03	0	0.00	0	0.00	2	0.03
V77I	Other	N	1	0.01	1	0.01	0	0.00	1	0.01
G73C	Accessory	Y	1	0.01	0	0.00	0	0.00	0	0.00
G73S	Accessory	Y	6	0.08	0	0.00	0	0.00	1	0.01
V82A	Major	Y	26	0.34	2	0.03	1	0.01	7	0.09
V82I	Other	N	32	0.42	7	0.09	10	0.13	88	1.16

PR	Classification	Surveillance	1-5%		5-10%		10-20%		>20%	
			n	%	n	%	n	%	n	%
V82F	Major	Y	4	0.05	0	0.00	0	0.00	1	0.01
V82L	Major	Y	0	0.00	0	0.00	1	0.01	1	0.01
V82T	Major	Y	1	0.01	0	0.00	0	0.00	0	0.00
N83D	Minor	Y	7	0.09	2	0.03	0	0.00	1	0.01
I84V	Major	Y	8	0.11	0	0.00	0	0.00	8	0.11
I85V	Other	N	10	0.13	1	0.01	3	0.04	17	0.22
N88D	Minor	Y	15	0.20	1	0.01	0	0.00	2	0.03
N88G	Major	N	0	0.00	0	0.00	0	0.00	15	0.20
N88S	Major	Y	10	0.13	1	0.01	0	0.00	0	0.00
N88T	Major	N	0	0.00	0	0.00	0	0.00	1	0.01
L89I	Other	N	0	0.00	0	0.00	0	0.00	2	0.03
L89M	Other	N	1	0.01	1	0.01	0	0.00	11	0.14
L89V	Minor	N	0	0.00	0	0.00	0	0.00	1	0.01
L90M	Major	Y	5	0.07	1	0.01	0	0.00	53	0.70

All drug resistance mutations reported. Analyses for 7614 individuals.

Table S6. Frequency of drug resistance mutations to NRTI at different sensitivity thresholds.

RT	Classification	Surveillance	1-5%		5-10%		10-20%		>20%	
			n	%	n	%	n	%	n	%
M41I	Other	N	29	0.38	2	0.03	0	0.00	34	0.45
M41L	NRTI	Y	12	0.16	2	0.03	0	0.00	59	0.77
E44D	NRTI	N	2	0.03	0	0.00	1	0.01	10	0.13
A62V	NRTI	N	4	0.05	0	0.00	1	0.01	12	0.16
K65E	NRTI	N	3	0.04	0	0.00	1	0.01	0	0.00
K65R	NRTI	Y	29	0.38	3	0.04	1	0.01	2	0.03
K65N	NRTI	N	0	0.00	1	0.01	0	0.00	0	0.00
A67N	Other	N	0	0.00	0	0.00	0	0.00	1	0.01
D67E	NRTI	Y	239	3.14	62	0.81	1	0.01	6	0.08
D67N	NRTI	Y	122	1.60	4	0.05	3	0.04	11	0.14
D67G	NRTI	Y	26	0.34	3	0.04	5	0.07	3	0.04
S68G	Other	N	1	0.01	5	0.07	3	0.04	123	1.62
T69A	Other	N	22	0.29	9	0.12	6	0.08	23	0.30
T69D	NRTI	Y	5	0.07	3	0.04	4	0.05	13	0.17
T69E	Other	N	3	0.04	0	0.00	0	0.00	0	0.00
T69I	Other	N	1	0.01	2	0.03	1	0.01	0	0.00
T69N	Other	N	211	2.77	35	0.46	8	0.11	22	0.29
T69S	Other	N	5	0.07	4	0.05	8	0.11	21	0.28
K70E	NRTI	Y	8	0.11	0	0.00	0	0.00	1	0.01
K70G	NRTI	N	0	0.00	0	0.00	0	0.00	1	0.01
K70N	NRTI	N	1	0.01	0	0.00	0	0.00	0	0.00
K70Q	NRTI	N	2	0.03	0	0.00	0	0.00	0	0.00
K70R	NRTI	N	16	0.21	2	0.03	3	0.04	7	0.09
K70T	NRTI	N	2	0.03	0	0.00	0	0.00	2	0.03
L74I	NRTI	Y	73	0.96	1	0.01	0	0.00	3	0.04
L74V	NRTI	Y	19	0.25	1	0.01	1	0.01	4	0.05
V75A	NRTI	Y	14	0.18	6	0.08	2	0.03	0	0.00
V75I	NRTI	N	19	0.25	1	0.01	0	0.00	9	0.12
V75L	NRTI	N	2	0.03	0	0.00	1	0.01	0	0.00
V75M	NRTI	Y	4	0.05	0	0.00	0	0.00	5	0.07
V75S	NRTI	N	4	0.05	0	0.00	0	0.00	1	0.01
V75T	NRTI	Y	0	0.00	0	0.00	0	0.00	1	0.01
F77L	NRTI	Y	138	1.81	1	0.01	1	0.01	5	0.07
V90I	Other	N	17	0.22	9	0.12	6	0.08	76	1.00
Y115F	NRTI	Y	1	0.01	0	0.00	0	0.00	4	0.05
F116Y	NRTI	N	1	0.01	0	0.00	0	0.00	1	0.01
M184V	NRTI	Y	36	0.47	5	0.07	3	0.04	21	0.28
M184I	NRTI	Y	30	0.39	5	0.07	3	0.04	10	0.13
M184V/I	NRTI	Y	23	0.30	2	0.03	0	0.00	4	0.05
L210F	Other	N	2	0.03	0	0.00	0	0.00	4	0.05
L210S	Other	N	0	0.00	0	0.00	0	0.00	5	0.07
L210W	NRTI	Y	8	0.11	3	0.04	1	0.01	18	0.24
T215A	NRTI	N	10	0.13	2	0.03	0	0.00	6	0.08
T215C	NRTI	Y	1	0.01	0	0.00	0	0.00	19	0.25
T215D	NRTI	Y	0	0.00	1	0.01	1	0.01	11	0.14
T215E	NRTI	Y	1	0.01	0	0.00	0	0.00	8	0.11
T215F	NRTI	Y	1	0.01	1	0.01	0	0.00	0	0.00
T215I	NRTI	Y	4	0.05	1	0.01	0	0.00	4	0.05
T215L	NRTI	N	0	0.00	0	0.00	0	0.00	4	0.05
T215N	NRTI	N	26	0.34	2	0.03	1	0.01	2	0.03
T215S	NRTI	Y	6	0.08	1	0.01	2	0.03	39	0.51
T215Y	NRTI	Y	3	0.04	0	0.00	1	0.01	1	0.01
K219E	NRTI	Y	9	0.12	4	0.05	1	0.01	7	0.09
K219N	NRTI	Y	4	0.05	2	0.03	0	0.00	9	0.12
K219Q	NRTI	Y	8	0.11	4	0.05	2	0.03	4	0.05

RT	Classification	Surveillance	1-5%		5-10%		10-20%		>20%	
			**n**	**%**	**n**	**%**	**n**	**%**	**n**	**%**
K219R	NRTI	Y	20	0.26	3	0.04	4	0.05	1	0.01

Analyses for 7614 individuals.

Table S7. Frequency of drug resistance mutations to NNRTI at different sensitivity thresholds.

RT	Classification	Surveillance	1-5%		5-10%		10-20%		>20%	
			n	%	n	%	n	%	n	%
A98G	NNRTI	N	1	0.01	0	0.00	0	0.00	8	0.11
L100I	NNRTI	N	26	0.34	1	0.01	0	0.00	5	0.07
L100V	NNRTI	N	2	0.03	0	0.00	0	0.00	0	0.00
K101E	NNRTI	Y	21	0.28	4	0.05	3	0.04	23	0.30
K101H	NNRTI	N	0	0.00	0	0.00	0	0.00	1	0.01
K101N	Other	N	1	0.01	0	0.00	0	0.00	0	0.00
K101P	NNRTI	Y	0	0.00	0	0.00	0	0.00	1	0.01
K101Q	Other	N	1	0.01	5	0.07	3	0.04	66	0.87
K101R	Other	N	4	0.05	1	0.01	1	0.01	11	0.14
K101T	Other	N	1	0.01	0	0.00	0	0.00	1	0.01
K103E	NNRTI	N	3	0.04	4	0.05	0	0.00	3	0.04
K103H	NNRTI	N	1	0.01	0	0.00	0	0.00	0	0.00
K103N	NNRTI	Y	20	0.26	17	0.22	15	0.20	246	3.23
K103Q	NNRTI	N	0	0.00	0	0.00	2	0.03	2	0.03
K103R	Other	N	21	0.28	9	0.12	9	0.12	162	2.13
K103S	NNRTI	Y	4	0.05	5	0.07	3	0.04	15	0.20
K103T	NNRTI	N	1	0.01	0	0.00	0	0.00	3	0.04
V106A	NNRTI	Y	11	0.14	4	0.05	5	0.07	5	0.07
V106I	Other	N	39	0.51	15	0.20	9	0.12	117	1.54
V106M	NNRTI	Y	2	0.03	0	0.00	1	0.01	3	0.04
V108I	NNRTI	N	29	0.38	7	0.09	10	0.13	40	0.53
V118I	Other	N	15	0.20	1	0.01	11	0.14	121	1.59
E138A	NNRTI	N	4	0.05	0	0.00	1	0.01	25	0.33
E138G	NNRTI	N	8	0.11	0	0.00	0	0.00	1	0.01
E138K	NNRTI	N	22	0.29	0	0.00	0	0.00	4	0.05
V179D	NNRTI	N	13	0.17	8	0.11	5	0.07	91	1.20
V179E	NNRTI	N	3	0.04	1	0.01	1	0.01	89	1.17
V179F	NNRTI	Y	1	0.01	0	0.00	0	0.00	2	0.03
V179I	NNRTI	N	29	0.38	24	0.32	10	0.13	316	4.15
V179L	NNRTI	N	0	0.00	0	0.00	0	0.00	2	0.03
V179T	NNRTI	N	4	0.05	2	0.03	2	0.03	6	0.08
Y181C	NNRTI	Y	11	0.14	8	0.11	4	0.05	33	0.43
Y188C	NNRTI	Y	14	0.18	8	0.11	1	0.01	3	0.04
Y181I	NNRTI	N	1	0.01	0	0.00	0	0.00	0	0.00
Y188H	NNRTI	Y	9	0.12	3	0.04	1	0.01	2	0.03
Y188L	NNRTI	Y	0	0.00	0	0.00	0	0.00	16	0.21
G190A	NNRTI	Y	7	0.09	6	0.08	10	0.13	37	0.49
G190E	NNRTI	Y	23	0.30	4	0.05	0	0.00	7	0.09
G190S	NNRTI	Y	3	0.04	0	0.00	0	0.00	3	0.04
G190V	NNRTI	N	1	0.01	0	0.00	0	0.00	0	0.00
H221Y	NNRTI	N	7	0.09	2	0.03	0	0.00	7	0.09
P225H	NNRTI	Y	133	1.75	5	0.07	3	0.04	19	0.25
F227C	NNRTI	N	9	0.12	0	0.00	0	0.00	0	0.00
F227L	NNRTI	N	79	1.04	3	0.04	2	0.03	2	0.03
M230I	NNRTI	N	10	0.13	2	0.03	1	0.01	0	0.00
M230L	NNRTI	Y	3	0.04	0	0.00	0	0.00	0	0.00
P236L	NNRTI	N	1	0.01	0	0.00	0	0.00	0	0.00
K238N	NNRTI	N	0	0.00	2	0.03	0	0.00	0	0.00
K238R	Other	N	27	0.35	11	0.14	5	0.07	13	0.17
K238T	NNRTI	N	1	0.01	0	0.00	1	0.01	5	0.07
Y318F	NNRTI	N	1	0.01	0	0.00	0	0.00	1	0.01
N348I	NNRTI	N	3	0.04	0	0.00	1	0.01	4	0.05

Table S8. Frequency of drug resistance mutations in integrase at different sensitivity thresholds.

IN	Classification	Surveillance	1-5%		5-10%		10-20%		>20%	
			n	%	n	%	n	%	n	%
H51Y	Accessory	N	5	0.12	0	0.00	0	0.00	0	0.00
T66A	Major	Y	4	0.10	1	0.02	1	0.02	0	0.00
T66I	Major	Y	6	0.14	2	0.05	2	0.05	1	0.02
T66K	Major	Y	164	3.95	0	0.00	0	0.00	0	0.00
L74I	Other	N	1	0.02	0	0.00	1	0.02	33	0.80
L74M	Other	N	1	0.02	1	0.02	0	0.00	5	0.12
L74V	Accessory	N	1	0.02	0	0.00	0	0.00	0	0.00
E92G	Major	N	7	0.17	1	0.02	0	0.00	0	0.00
E92V	Major	N	1	0.02	0	0.00	0	0.00	0	0.00
E92Q	Major	Y	1	0.02	0	0.00	0	0.00	0	0.00
Q95K	Accessory	N	34	0.82	0	0.00	0	0.00	7	0.17
T97A	Accessory	N	12	0.29	1	0.02	2	0.05	13	0.31
G118R	Major	N	0	0.00	1	0.02	0	0.00	0	0.00
F121Y	Major	Y	1	0.02	0	0.00	0	0.00	0	0.00
A128T	Accessory	N	11	0.27	5	0.12	3	0.07	3	0.07
E138D	Other	N	11	0.27	5	0.12	5	0.12	124	2.99
E138K	Major	N	14	0.34	9	0.22	3	0.07	4	0.10
E138A	Major	N	1	0.02	1	0.02	0	0.00	10	0.24
G140C	Major	Y	2	0.05	0	0.00	0	0.00	0	0.00
G140S	Major	Y	3	0.07	2	0.05	0	0.00	0	0.00
Y143C	Major	Y	5	0.12	0	0.00	1	0.02	0	0.00
Y143H	Major	Y	5	0.12	0	0.00	0	0.00	0	0.00
P145S	Major	N	5	0.12	0	0.00	0	0.00	0	0.00
Q146P	Major	N	6	0.14	0	0.00	0	0.00	0	0.00
S147G	Major	Y	14	0.34	2	0.05	1	0.02	0	0.00
Q148H	Major	Y	2	0.05	0	0.00	0	0.00	1	0.02
Q148K	Major	Y	25	0.60	0	0.00	0	0.00	0	0.00
Q148R	Major	Y	7	0.17	1	0.02	1	0.02	1	0.02
V151A	Accessory	N	3	0.07	1	0.02	1	0.02	0	0.00
V151I	Other	N	24	0.58	8	0.19	4	0.10	19	0.46
S153F	Accessory	N	2	0.05	1	0.02	2	0.05	0	0.00
S153Y	Accessory	N	25	0.60	0	0.00	0	0.00	0	0.00
N155H	Major	Y	2	0.05	0	0.00	0	0.00	0	0.00
N155S	Major	Y	3	0.07	0	0.00	0	0.00	0	0.00
N155T	Major	N	0	0.00	1	0.02	0	0.00	0	0.00
E157Q	Accessory	N	4	0.10	2	0.05	2	0.05	17	0.41
G163K	Accessory	N	1	0.02	1	0.02	0	0.00	4	0.10
G163R	Accessory	N	12	0.29	1	0.02	1	0.02	2	0.05
S230N	Other	N	26	0.63	6	0.14	13	0.31	436	10.51
S230R	Accessory	N	130	3.13	3	0.07	1	0.02	2	0.05
R263K	Accessory	N	5	0.12	1	0.02	0	0.00	1	0.02

Analyses for 4148 individuals.
